# Narrow-Energy-Width CT Based on Multivoltage X-Ray Image Decomposition

**DOI:** 10.1155/2017/8126019

**Published:** 2017-11-07

**Authors:** Jiaotong Wei, Yan Han, Ping Chen

**Affiliations:** Shanxi Key Laboratory of Signal Capturing & Processing, North University of China, Taiyuan 030051, China

## Abstract

A polychromatic X-ray beam causes the grey of the reconstructed image to depend on its position within a solid and the material being imaged. This factor makes quantitative measurements via computed tomography (CT) imaging very difficult. To obtain a narrow-energy-width reconstructed image, we propose a model to decompose multivoltage X-ray images into many narrow-energy-width X-ray images by utilizing the low frequency characteristics of X-ray scattering. It needs no change of hardware in the typical CT system. Solving the decomposition model, narrow-energy-width projections are obtained and it is used to reconstruct the image. A cylinder composed of aluminum and silicon is used in a verification experiment. Some of the reconstructed images could be regarded as real narrow-energy-width reconstructed images, which demonstrates the effectiveness of the proposed method.

## 1. Introduction

With the development and application of advanced technology, computed tomography (CT) has changed from conventional qualitative imaging for detection to quantitative functional imaging for distinguishing and identifying different components. For instance, quantifying the composition of coal and the microstructure of mineral grain contributes to an understanding of the transformation of minerals during coal processing, which promotes the development of clean coal technologies [1]. Quantifying the three-dimensional microstructure of excipients contributes to the development and testing of new drugs [2]. Quantification of soil aggregate microstructure on abandoned cropland during vegetative succession allows determination of the retention and transport of water, gases, and nutrients in soils, thus allowing preservation of soil productivity and maintaining soil porosity and resistance to erosion [3]. In these applications, good congruity is needed between the linear attenuation coefficient and X-ray energy in the reconstructed images. In other words, the linear attenuation coefficient of the same component should be uniform, and the corresponding energy of different components' linear attenuation coefficients should be uniform in a single reconstructed image. A higher grey value corresponds to a larger linear attenuation coefficient in one reconstructed image. It is polychromatic X-ray in the typical CT system and leads to cupping artifacts, which is that the grey of the reconstructed image depends on both the material and its position [4, 5]. So if two materials have approximately linear attenuation coefficients, their grey may overlap, which makes them difficult to distinguish. Overlapping attenuation coefficients makes quantitative imaging very challenging. The use of monochromatic radiation can eliminate cupping artifacts and accomplish a one-to-one relationship between grey values and materials [6]. But it is impractical to apply monochromatic radiation in the typical CT system [7, 8]. One feasible method is to synthesize monochromatic images using dual-energy imaging. One example is the gemstone spectral imaging (GSI) systems. It is a type of dual-energy CT and its X-ray is polychromatic [9]. In the synthesized monochromatic images, the CT numbers become more accurate, but they are still not truly monochromatic, especially at low energy [10]. Another feasible method is to obtain narrow-energy-width images, which can approximate monochromatic images. It can be accomplished through multienergy imaging, which may require an X-ray photon counting detector [11, 12]. Multienergy imaging can be seen an extension of dual-energy CT [7, 13]. The photon counting detector can count discrete photon interactions [14] and has energy selectivity. So it can improve contrast in CT and apply to the material identification [15]. The imaging system based on photon counting detector shows effectiveness in distinguishing different materials [16]. Photon counting detectors are used in nuclear medicine and spectral mammography, but they are not commercially available for CT systems [14]. Challenges remain for them since the exposure rates are insufficient when used to CT imaging [14].

The photon counting detector can obtain many narrow-energy-width images by selecting energy bands. A multienergy CT imaging method was presented based on energy spectrum filtering separation [17], which can, in theory, distinguish different components. However, the application of the multienergy CT imaging method is limited in practice because the energy spectrum can only be narrowed to maintain X-ray penetrability, and the difference between different spectra is insufficient. Another method to obtain narrow-energy-width images is to decompose the multivoltage X-ray images acquired in a typical CT system [18]. This can improve the contrast of different materials with approximately linear attenuation coefficients in reconstructed images [18]. However, these reconstructed images are not real narrow-energy-width reconstructed images, as their contrast is much larger than the theoretical value [18].

Building on previous research [18], we continued studying the decomposition approach of multivoltage X-ray images to obtain a narrow-energy-width projection with a typical CT system without changes in hardware. Herein, we present a new decomposition model based on X-ray scattering characteristics. Some reconstructed images obtained with the new decomposition model can be regarded as real narrow-energy-width reconstructed images. The remainder of this paper is organized as follows. In Section 2, the decomposition model of multivoltage X-ray images presented in [18] is introduced. In Section 3, the new decomposition model and its solution algorithm are presented. Then, in Section 4, the new method is applied to obtain the narrow-energy-width reconstructed image of a cylinder composed of aluminum and silicon. In Section 5, the discussion of innovations and shortcomings of the method are presented, along with upcoming work. Finally, our conclusions are presented.

## 2. Previous Decomposition Model of Multivoltage X-Ray Images

The X-ray emitted from an X-ray tube is polychromatic and can be split into many narrow-energy-width bands. Therefore, a polychromatic X-ray image can be seen as the sum of many narrow-energy-width X-ray images. The X-ray imaging can be described as follows:

(1) $$\begin{array}{c} I=I_{0}\overset{R}{\underset{r=1}{\sum }}S\left(\;E_{r}\;\right)\exp\left(\;-\overset{K}{\underset{k=1}{\sum }}u_{rk}d_{k}\;\right), \end{array}$$

where *I*_0_ is initial X-ray intensity, *I* is final X-ray intensities, *r* means different narrow energy bands, *k* denotes different materials, *E*_*r*_ is the energy of the *r*th narrow energy band, *S*(*E*_*r*_) means the weighted coefficient of the *r*th narrow-energy-width X-ray image, *u*_*rk*_ is a linear attenuation coefficient depending on the *k*th material being traversed by the X-ray and the energy level of the *r*th narrow-energy-width band, and the distance the X-ray traverses through the *k*th material is denoted as *d*_*k*_ [18]. *S*(*E*_*r*_) is related to the incident X-ray spectrum and the detector efficiency:

(2) $$\begin{array}{c} 1=\overset{R}{\underset{r=1}{\sum }}S\left(\;E_{r}\;\right). \end{array}$$

The weighted coefficients *S*(*E*_*r*_) are unknown because the energy spectrum is unknown. The narrow-energy-width X-ray images can be get from the decomposition of multiple X-ray images with different voltages [18]. In other words, the narrow-energy-width projection can be obtained and can be used to reconstruct a narrow-energy-width CT image.

*I*/*I*_0_ of the *m*th pixel in the X-ray image of *n*th voltage is denoted as *f*_*nm*_, *n* = 1,2,…, *N*, *m* = 1,2,…, *M*, and the multivoltage X-ray imaging model is

(3) $$\begin{array}{c} F=S\exp\left(\;-UD\;\right)+\Delta F, \end{array}$$

where *F* = (*f*_*nm*_)_*NM*_, *S* = (*s*_*nr*_)_*NR*_, *U* = (*u*_*rk*_)_*RK*_, *D* = (*d*_*km*_)_*KM*_, Δ*F* = (Δ*f*_*nm*_)_*NM*_, and Δ*F* is the error produced by measurement and scattering [19–21]. The *n*th row of *S* is the weighted coefficients of the narrow-energy-width X-ray images to constitute the X-ray image at the *n*th voltage. The value of *d*_*km*_ is the *m*th pixel's corresponding distance that the X-ray traversed through the *k*th material. When several materials are uniformly mixed, they are considered one material [18]. To guarantee that the information related to narrow-energy-width X-ray images is sufficient, *N*, *M*, *R*, and *K* should satisfy the following inequality [18]:

(4) $$\begin{array}{c} NM>NR+RK+KM. \end{array}$$

The solution is translated to a least squares optimization model as

(5) min F−Sexp⁡−UDFF2s.t. S≥0, U≥0, D≥0.

This model can be solved with the Karush-Kuhn-Tucker (KKT) condition [18]. In the verification experiment [18], the materials with approximately linear attenuation coefficients in the reconstructed images could be significantly distinguished. However, the contrast of the materials is larger than it should be in a real narrow-energy-width reconstructed image. In other words, the reconstructed images are not real narrow-energy-width reconstructed images. This may be because scattering is not considered in model (5).

## 3. Decomposition Model Based on X-Ray Scattering Character

During X-ray imaging, scattering is an important interference factor, especially when a flat panel detector is used [22]. Significant research on scattering is available. From [22–25], scattering is a low frequency signal related to the imaging objects. The estimated scattering is obtained by multiplying a coefficient to the low-pass filter of the original image, and the scattering suppression method is the original image minus the estimated scattering. This method was quite effective in [23, 25]. In the X-ray imaging model in [26], scattering is regarded as a constant over the entire projection and the same for all projections and depends on the object in the scan. Summarizing the aforementioned research results, scattering is a low frequency signal.

A low frequency signal indicates slow change. In other words, the difference of the neighboring sampling nodes is small; therefore, variance is used to describe this characteristic. Because scattering is related to the imaging object, different projections may result in different scattering. For this reason, the local variance of a signal sampling node is used to estimate the change rate of the sampling node. The whole scattering character is described with the sum of all local variance. The initial intensity of the X-ray beam is greater than 1, so the signal of dividing scattering by *I*_0_ is also a low frequency signal. The decomposition model of multivoltage X-ray images can be considered:

(6) min G=∑n=1 N∑m=T+1M−TvarF−Sexp⁡−UDnms.t. S≥0, U≥0, D≥0,

where *T* is a parameter related to local image size (and it needs to set up first in order to solve the model) and *nm* is the local image whose center is the *m*th pixel in the X-ray image of *n*th voltage. For example, the local image size is 1 × (2*T* + 1), and the current pixel is the center in the 2-dimensional CT reconstruction. To reconstruct an image, the projections of many different angles are needed; then formula (6) is changed as

(7) min G=∑n=1 N∑j=1 J∑m=T+1Mj−TvarF−Sexp⁡−UDnjm

(8)  U≥0,

(9)  D≥0,

where *j* denotes different angles; *n*(*jm*) means the local image, whose center is the *m*th pixel in the *j*th angle X-ray image of *n*th voltage; and *M*_*j*_ denotes the pixel amount in the *j*th angle X-ray image.

Similar to [18], formula (7) can be solved by the KKT condition. The iterative formulas are

(10) S=S⊙SupSdown

(11) U=U⊙UupUdown

(12) D=D⊙DupDdown

(13) Sup=2Se−UD⊗11×2T+1⊙OBN×2T+1∑g=1JMge−UD⊗11×2T+1T2T+1+FOTe−UDOTT2T+12

(14) Sdown=2F⊗11×2T+1⊙OBN×2T+1∑g=1JMge−UD⊗11×2T+1T2T+1+2Se−UDOTe−UDOTT2T+12

(15) Uup=2STSe−UD⊗11×2T+1⊙OBR×2T+1∑g=1JMg⊙e−UD⊗11×2T+1D⊗11×2T+1T2T+1+22T+12∑t=−TTSTFOT⊙e−UD→tD→tT

(16) Udown=2STF⊗11×2T+1⊙OBR×2T+1∑g=1JMg⊙e−UD⊗11×2T+1D⊗11×2T+1T2T+1+22T+12∑t=−TTSTSe−UDOT⊙e−UD→tD→tT

(17) Dup=22T+1UTSTSe−UD⊙OD⊙e−UD+22T+12UTSTFO2T⊙e−UD

(18) Ddown=22T+1UTSTF⊙OD⊙e−UD+22T+12UTSTSe−UDO2T⊙e−UD,

where “⊙” is the Hadamard product. 1_1×(2*T*+1)_ means a matrix with every element = 1 with 1 row and (2*T* + 1) columns. “( )_→*t*_” means the matrix moves left *t* columns (right if the *t* is negative), and the empty columns at the boundary are replaced with original columns. *O*_*T*_ means a matrix with ∑_*j*=1_^*J*^*M*_*j*_ rows and ∑_*j*=1_^*J*^*M*_*j*_ columns, and only the element at the row from *m* − *T* + ∑_*g*=1_^*j*−1^*M*_*g*_ to *m* + *T* + ∑_*g*=1_^*j*−1^*M*_*g*_ in *m* + ∑_*g*=1_^*j*−1^*M*_*g*_  (*m* = *T* + 1, *T* + 2,…, *M*_*j*_ − *T*) column is 1, while the others are 0. *O*_2*T*_ means a matrix with ∑_*j*=1_^*J*^*M*_*j*_ rows and ∑_*j*=1_^*J*^*M*_*j*_ columns, and the *m* + ∑_*g*=1_^*j*−1^*M*_*g*_  (*m* = 2*T* + 1,2*T* + 2,…, *M*_*j*_ − 2*T*) column is

(19) O2T:m+∑g=1j−1Mg=0⋯012⋯2T2T+12T⋯210⋯0T,

where the nonzero row is from *m* − 2*T* + ∑_*g*=1_^*j*−1^*M*_*g*_ to *m* + 2*T* + ∑_*g*=1_^*j*−1^*M*_*g*_, *m* + ∑_*g*=1_^*j*−1^*M*_*g*_  (*m* = 1,2,…, 2*T*), and the column is

(20) O2T:m+∑g=1j−1Mg=12⋯mmm⋯210⋯0T,

where the nonzero row is from 1 + ∑_*g*=1_^*j*−1^*M*_*g*_ to *m* + 2*T* + ∑_*g*=1_^*j*−1^*M*_*g*_, *m* + ∑_*g*=1_^*j*−1^*M*_*g*_  (*m* = *M*_*j*_ − 2*T* + 1, *M*_*j*_ − 2*T* + 2,…, *M*_*j*_), and the column is

(21) $$\begin{array}{c} {\left(\;O_{2T}\;\right)}_{:\left(m+\sum_{g=1}^{j-1}M_{g}\right)}={\left(\;\begin{array}{cccccccccccc} 0 & \cdots & 0 & 1 & 2 & \cdots & M_{j}+1-m & M_{j}+1-m & M_{j}+1-m & \cdots & 2 & 1 \end{array}\;\right)}^{T}, \end{array}$$

where the nonzero row is at *m* − 2*T* + ∑_*g*=1_^*j*−1^*M*_*g*_ to *M*_*j*_ + ∑_*g*=1_^*j*−1^*M*_*g*_. *O*_*B*(*x* × (2*T* + 1)(∑_*j*=1_^*J*^*M*_*j*_))_ means a matrix with *x* rows and (2*T* + 1)(∑_*j*=1_^*J*^*M*_*j*_) columns and all elements equal to 1 when the column is from 1 + (2*T* + 1)(*m* − 1)+(2*T* + 1)∑_*g*=1_^*j*−1^*M*_*g*_ to (2*T* + 1)*m* + (2*T* + 1)∑_*g*=1_^*j*−1^*M*_*g*_  (*j* = 1,2,…, *J*, *m* = 2*T* + 1,2*T* + 2,…, *M*_*j*_ − 2*T*), from 1 + (2*T* + 1)(*m* − 1)+(2*T* + 1)∑_*g*=1_^*j*−1^*M*_*g*_ to *m* + (2*T* + 1)(*m* − 1)+(2*T* + 1)∑_*g*=1_^*j*−1^*M*_*g*_  (*j* = 1,2,…, *J*, *m* = 1,2,…, 2*T*), from 2*T* + 1 − (*M*_*j*_ − *m*)+(2*T* + 1)(*m* − 1)+(2*T* + 1)∑_*g*=1_^*j*−1^*M*_*g*_ to (2*T* + 1)*m* + (2*T* + 1)∑_*g*=1_^*j*−1^*M*_*g*_  (*j* = 1,2,…, *J*, *m* = *M*_*j*_ − 2*T* + 1, *M*_*j*_ − 2*T* + 2,…, *M*_*j*_). All elements of other columns are 0. *O*_*D*_ means a matrix with *R* rows and ∑_*j*=1_^*J*^*M*_*j*_ columns where every column of *m* = 2*T* + 1,2*T* + 2,…, *M*_*j*_ − 2*T* is as ${\left(O_{D}\right)}_{:column}=(2T+1){\left(\begin{array}{ccccc} 1 & \cdots & 1 & \cdots & 1 \end{array}\right)}^{T}$; every column of *m* = 1, 2,…, 2*T* is ${\left(O_{D}\right)}_{:column}=m{\left(\begin{array}{ccccc} 1 & \cdots & 1 & \cdots & 1 \end{array}\right)}^{T}$; every column of *m* = *M*_*j*_ − 2*T* + 1, *M*_*j*_ − 2*T* + 2,…, *M*_*j*_ is ${\left(O_{D}\right)}_{:column}=(M_{j}+1-m){\left(\begin{array}{ccccc} 1 & \cdots & 1 & \cdots & 1 \end{array}\right)}^{T}$.

Similar to [18], the multiplicity solution of *U* and *D* still exists due to putting a pair invertible matrix between *U* and *D*. As the eventual goal is the product* UD*, we considered that they are the same solution. Every row of *S* is normalized according to (2). The following is the complete algorithm to solve (7):

(1) initialize *S*, *U*, *D*;
(2) set maximum number of iterations* niter* and a small value *ε*;
(3) ** for ***niter* = 1,2,…, *niter*,
  (a) update *S* according to (10);
  (b) normalize every row of *S* with
    (22) $$\begin{array}{c} s_{nr}=\frac{s_{nr}}{\sum_{r=1}^{R}s_{nr}} \end{array}$$
  (c) update *U* according to (11);
  (d) update *D* according to (12);
  (e) compute the value of the objective function of (7), and note as* y*;
  (f) ** if ***y* < *ε*,
  iteration terminates
  **end**
**end**

The solution may be a local minimum, so the algorithmic processes must be repeated many times with different initializations. The optimal solution is selected from the many results.

## 4. Results

**Figure 1:**
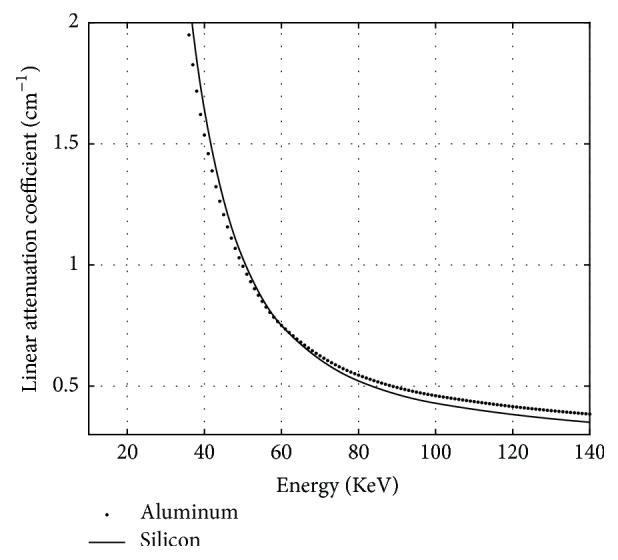
Linear attenuation coefficients of aluminum and silicon.

A cylinder made of aluminum and silicon was used in the verification experiment because the two materials' linear attenuation coefficients are approximate. The linear attenuation coefficients of aluminum and silicon are near-equal at approximately 60 KeV, and, from 10 to 140 KeV, their max difference is less than 13%, as shown in Figure 1. Thus, for the contrast of aluminum and silicon in the reconstructed image, the absolute value should first decrease and then increase as the voltage increases from 10 KeV. The linear attenuation coefficient was obtained from National Institute of Standards and Technology (NIST), and the values were processed using cubic spline interpolations. Some origin values of NIST and difference of the linear attenuation coefficient of aluminum and silicon are shown in Table 1. The silicon was on the outside, and the aluminum was on the inside of the cylinder. The cylinder's diameter was 40 mm, and the aluminum's diameter was 30 mm.

In the experiment, an X-ray source (ISOVOLT TITAN 4503PH with 450/5 tube housing) was operated at a tube current of 3 mA and tube voltages of 120, 130, and 140 kV. The applied flat panel detector (PerkinElmer XRD1621 AN14 ES) was 2,048 × 2,048 cells of size 0.2 mm, and only a portion of a row of data was used for 2D image reconstruction. The source-object distance (SOD) was 120 cm, and the object-detector distance (ODD) was 20 cm. The angular sampling interval was 2 degrees, and the data were obtained for 180 angles. The image reconstruction algorithm was an algebraic reconstruction algorithm (ART), and the contribution coefficient of every pixel was the distance the X-ray traversed through the pixel. The reconstructed images had some noise and were denoised with a median filter with a window size of 5 × 5.

**Figure 2:**
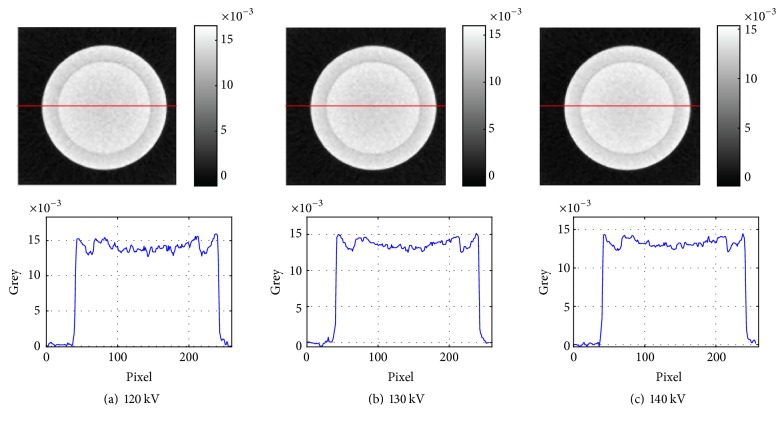
Direct reconstructed images.

The direct reconstructed images of 120, 130, and 140 kV are shown in Figure 2.

**Figure 3:**
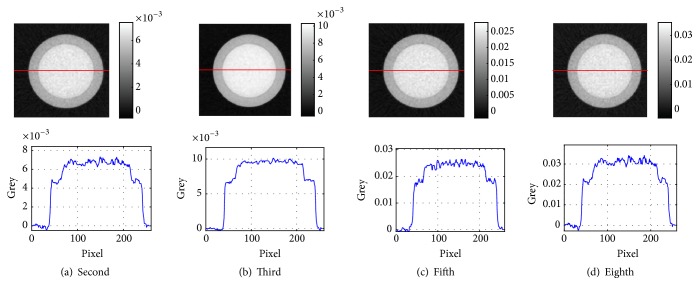
Four representational reconstructed images obtained by the method of [18].

Four representational reconstructed images with the lowest noise were selected from the results obtained by the method in [18] and are shown in Figure 3.

**Figure 4:**
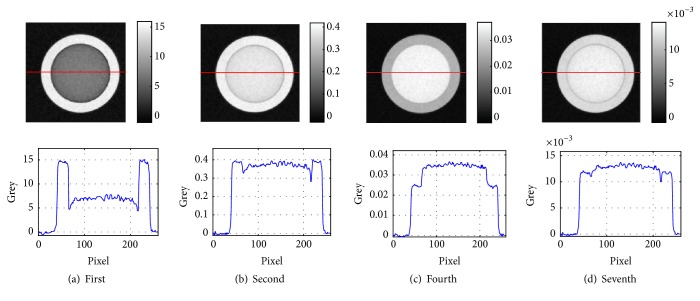
Four representational reconstructed images obtained by the method of this paper.

To decompose the multivoltage X-ray images, we used the method of this paper. The X-ray images were decomposed by the proposed method with *R* = 14. The last two coefficients of the row of *S*, corresponding to 120 kV, were set to zero. The last coefficient of the row of *S*, corresponding to 130 kV, was set to zero. These coefficients are the same as those in [18] with the empirical parameter *T* = 20. To decrease the iterative time, a good initial value of *S*, *U*, and *D* were given. The estimated *D* can be computed by combining the threshold segmentation of the image (Figure 3(b)). The estimated *S* can be computed by normalizing the simulation energy spectrum, which comes from the simulation software Spectrum GUI_1.03. The estimated *U* can be replaced with the linear attenuation coefficient of center energy at every energy interval. The initial values of *S*, *U*, and *D* were selected as random increases or decreases less than 10% based on their estimation. Since the iterative updating formula is a multiplicative model, a small value of 0.001 was added to the initial value of *D* to avoid that 0 always is 0. The max iterative time was 500. The stopping condition was the difference of neighboring two objective function values less than *ε* = 0.1%. The optimal objective function value was 1.1377, which was obtained with many repetitions. Four representational reconstructed images were selected from the results and are shown in Figure 4.

Since the result images of the method in [18] are out-of-order, which is influenced by its initial value, and some images of them with high noise level have very poor image quality and the sequence numbers of selected images are different in Figures 3 and 4.

The cupping artifact, which is caused by beam hardening, is an important characteristic of polychromatic reconstructed images. The cupping artifact is apparent in the reconstructed images in Figure 2 and causes the greys of aluminum and silicon to overlap. Compared to the reconstructed images in Figure 2, the cupping artifacts in the reconstructed images of Figures 3 and 4 apparently weaken, which conforms to narrow-energy-width reconstructed images.

We further verified whether the contrast of these reconstructed images matched the narrow-energy-width characteristics. From Figure 1, the greys of narrow-energy-width reconstructed images can be classified into three types: grey of silicon larger than that of aluminum at low X-ray energy, grey of silicon close to that of aluminum at middle X-ray energy, and grey of silicon smaller than that of aluminum at high X-ray energy. Comparing the reconstructed images in Figures 3 and 4, the images in Figure 4 are more consistent with this change. To compute the contrast of aluminum and silicon in the reconstructed images of Figures 3 and 4, we used the following formula:

(23) $$\begin{array}{c} c_{\text{contrast}}=\frac{g_{Al}-g_{Si}}{g_{Al}}, \end{array}$$

where *g*_*Al*_ is the average grey of the aluminum region and *g*_*Si*_ is the average grey of the silicon region. The results from Figure 3 are presented in Table 2, and the results from Figure 4 are presented in Table 3.

In Table 2, the contrast of all reconstructed images was much higher than 13%, which implies that these reconstructed images were not real narrow-energy-width reconstructed images. This finding is consistent with the conclusion of [18].

In Table 3, the contrast of the first and fourth reconstructed images was also much higher than 13%, which implies that they are not real narrow-energy-width reconstructed images. However, the second and seventh reconstructed image contrast was within the realm of theory. In the ART, the distance unit or length unit is pixel and the pixel size is equal to it of X-ray images. By computing their linear attenuation coefficients, the method implies that the grey should be multiplied by 5 since the detector cell size was 0.2 mm if the unit of linear attenuation coefficient is mm^−1^. The linear attenuation coefficient of aluminum and silicon was close to 16 KeV in the second reconstructed image. The linear attenuation coefficient of aluminum was close to 69 KeV, and the silicon was close to 73 KeV in the seventh reconstructed image. The energy difference between aluminum and silicon was acceptable if the two reconstructed images are regarded as narrow-energy-width reconstructed images. In other words, the narrow-energy-width reconstructed images are produced by the decomposition of multivoltage X-ray images when using the method described in this paper.

## 5. Discussion

The proposed method can be regarded as in-depth research of the concept that is presented in [18] to obtain narrow-energy-width reconstructed images in the typical CT imaging system without changing hardware. Compared to the previous multivoltage X-ray image decomposition model, the new decomposition model considers the influence of X-ray scattering. Scattering is an important factor that disturbs the accuracy of X-ray imaging. Scattering is a nonnegative value for whole X-ray imaging. Thus, the error caused by scattering is not suitably described with the weighted least square in [18]. The low frequency characteristic of scattering is embedded in the new decomposition model and should be more reasonable than the previous model. This assumption is validated in the verification experiment, where no reconstructed image was a real narrow-energy-width reconstructed image by solving with the previous model; there are some reconstructed images that can be seen as real narrow-energy-width reconstructed images by solving with the model in this paper. The proposed method provides a glimmer of light by obtaining narrow-energy-width reconstructed images with a typical CT imaging system without changing hardware and knowing the energy spectrum, which is difficult to accurately measure. This may improve the application potential of typical CT imaging systems, whereas a monochromatic or narrow-energy-width X-ray source and photon counting detector are expensive.

However, many reconstructed images obtained with the new decomposition model are still not real narrow-energy-width reconstructed images. This implies that the new decomposition model is still imperfect. The variance description for the low frequency characteristic of scattering is rough. Apparently, the optimal solution for the new decomposition model is that the scattering is constant throughout the whole X-ray image; this solution is unrealistic because of the complicacy of scattering. However, X-ray imaging errors are not all caused by scattering. Therefore, a more accurate model of practical X-ray imaging is needed to obtain a more accurate narrow-energy-width projection.

In addition to optimizing the decomposition model, two other problems require further research. One is the selection of parameter *T*, which was empirically selected in this paper. However, *T* is not a strict value because low frequency yields a blurry description. The second issue is to improve the new model's present solving algorithm, which may converge to a local minimum solution and is of slow convergence.

Furthermore, there is nowhere to need axisymmetric structure in the method. The cylinder has axisymmetric structure, but its center is not the center of CT scan, which can be observed from the reconstructed images. This can partly show that no relationship between the shape of materials and the method. Similarly, there is no relationship between the energy range of X-ray and the method, since nowhere needs special energy range of X-ray.

The noise is another problem that needs careful attention. In this paper, there is no special denoise processing for the origin data. In other words, the method of this paper is effective when there is general noise. From the model, the residual error of X-ray images decomposition is constant when the solution is ideal optimal, since the variance will be zero. Then the noise will be shared by the narrow-energy-width X-ray images. So it is foreseeable that the method is affected by the noise and the method may be invalid if the noise is too larger. And the further conclusion needs more research.

## 6. Conclusion

In conclusion, we have proposed a novel multivoltage X-ray image decomposition model to obtain narrow-energy-width projections based on the low frequency characteristics of scattering in X-ray imaging without changing the existing hardware. The verification experiment shows that some reconstructed images obtained with this model are completely in conformity with narrow-energy-width reconstructed images. Further work is under way, including optimization of the decomposition model and algorithm.

## Figures and Tables

**Table 1:** Some origin values of NIST and difference of the linear attenuation coefficient of aluminum and silicon.

Energy/KeV	10.32	30.01	51.19	62.53	71.46	93.31	113.99	130.27
Aluminum/cm^−1^	62.32	3.013	0.9526	0.7119	0.6112	0.4876	0.4318	0.4038
Silicon/cm^−1^	69.46	3.293	0.9693	0.6984	0.5865	0.4519	0.3933	0.3648
Difference	−11.46%	−9.29%	−1.75%	1.90%	4.04%	7.32%	8.92%	9.66%

**Table 2:** Contrast of aluminum and silicon in the reconstructed images from Figure 3.

Image	(a) second	(b) third	(c) fifth	(d) eighth
Contrast	0.2789	0.2759	0.2718	0.2797

**Table 3:** Contrast of aluminum and silicon in the reconstructed images from Figure 4.

Image	(a) first	(b) second	(c) fourth	(d) seventh
Contrast	−1.1290	−0.0442	0.2870	0.0871
